# Extracting h-Backbone as a Core Structure in Weighted Networks

**DOI:** 10.1038/s41598-018-32430-1

**Published:** 2018-09-25

**Authors:** Ronda J. Zhang, H. Eugene Stanley, Fred Y. Ye

**Affiliations:** 1 Jiangsu Key Laboratory of Data Engineering and Knowledge Service, School of Information Management, Nanjing University, Nanjing, 210023 China; 2 International Joint Informatics Laboratory, University of Illinois at Urbana-Champaign, USA and Nanjing University, Nanjing, China; 3 Department of Physics and Center for Polymer Studies, Boston University, Boston, Massachusetts 02215 USA

## Abstract

Determining the core structure of complex network systems allows us to simplify them. Using *h-*bridge and *h*-strength measurements in a weighted network, we extract the *h-*backbone core structure. We find that focusing on the *h*-backbone in a network allows greater simplification because it has fewer edges and thus fewer adjacent nodes. We examine three practical applications: the co-citation network in an information system, the open flight network in a social system, and coauthorship in network science publications.

## Introduction

The contemporary study of complex networks began with Watts & Strogatz^1^ and Barabási & Albert^2^, and the resulting complex network science is now widely used in research on social, information, biological, and technological networks^3–9^. Although extracting the network backbone is an important task in network analysis^10–12^, it is difficult to extract the interactions between nodes or edges and the unique core structure. The numerous attempts to extract the backbone of a complex network have used different values—e.g., the degree distribution or the edge-betweenness centrality distribution^13^—in an effort to preserve backbone information. Other approaches have focused on network type—e.g., economic systems^14^ or online recommendation networks^15^. Another key issue is that backbones are not unique, and some parameters need an artificial setting.

Using the *h*-index^16^ metric, which is now commonly used in recommendation networks and its other network applications^17^, we introduced *h*-degree and *h*-strength and extracted the ***h-***core and *h*-subnet of a weighted network^18–20^. Although in this work we were able to use *h*-degree and *h*-strength factors to extract functionally significant core information, we note that both *h*-factors overlook nodes and edges that have a relatively low weight—the very network nodes and edges often vital in transporting the flow of information. Also, according to the weak tie theory^21,22^, we notice that some weak links can be structurally important in networks.

To quantify the importance of each node and edge in a given network, since the 1970s, different types of centralities have been defined^23–26^. When extracting important network information, ranking edge centrality is more effective than ranking node centrality. This is because nodes can exist in isolation, but edges always connect two nodes. Edge weights are naturally generated in a network, better represent interaction levels between nodes, and thus provide an index that quantifies the importance of network functions. At the same time, edge betweenness reveals the structural characteristics of a network. In some of the literature^13,27^ edge betweenness is used to extract the structural skeleton of a network. Thus combining edge weight and edge betweenness can provide important information about both network function and structure.

In our research we combine the *h*-bridge and *h*-strength to capture the structurally important interactions of edges with adjacent nodes. After extracting the structural *h*-bridge and the functional *h*-strength in a weighted network, we synthesize an *h*-backbone that combines both structural and functional interactions.

## Data

We use three sets of data in our research.

1. **Co-citation network**: From the ISI Web of Science (WoS) on 18 May 2017 we obtained the top 100 most-cited articles that cited Hirsch’s original paper that defined the *h-*index (“An index to quantify an individual’s scientific research output”). We examined the references that occurred more than five times, set up a co-citation network, and then deleted Hirsch’s original paper. Allowing it to remain would have affected the edge betweenness because it was connected to all the other references.
2. **Open flight network**: We obtained the updated open flight data online in January 2012 (https://openflights.org/data.html). It lists approximately 60,000 routes between over 3200 airports worldwide. We transformed the data into an undirected weighted network in which the weight of a route is the number of airlines flying between two nodes (two airports).
3. **Coauthorship in network science publishing**: We also use classic coauthorship network of scientists working on network theory and experiment^24^ compiled by M. Newman in May 2006. We assign the network weights as described in Newman’s work^26^.
4. These three data sets represent two typical networks. The first and the last are information networks, and the second a social (transportation) network. Table 1 shows the main features of these weighted networks.

**Table 1:** The sample data with network parameters.

Parameters	Co-citation network	Open flight network	Coauthorship network
number of nodes	566	3425	1589
number of edges	31,337	19,256	2742
*h*-bridge	11	23	8
number of edges of *h*-bridge	11	23	3
*h*-strength	12	20	3
number of edges of *h*-strength	15	33	8

The number of edges with weights higher than or equal to the *h*-strength is greater than the *h*- strength because there are many nodes with weights identical to the *h*-strength.

## Results

We run experiments to test our method of identifying the *h*-backbone in a weighted network.

**Figure 1:**
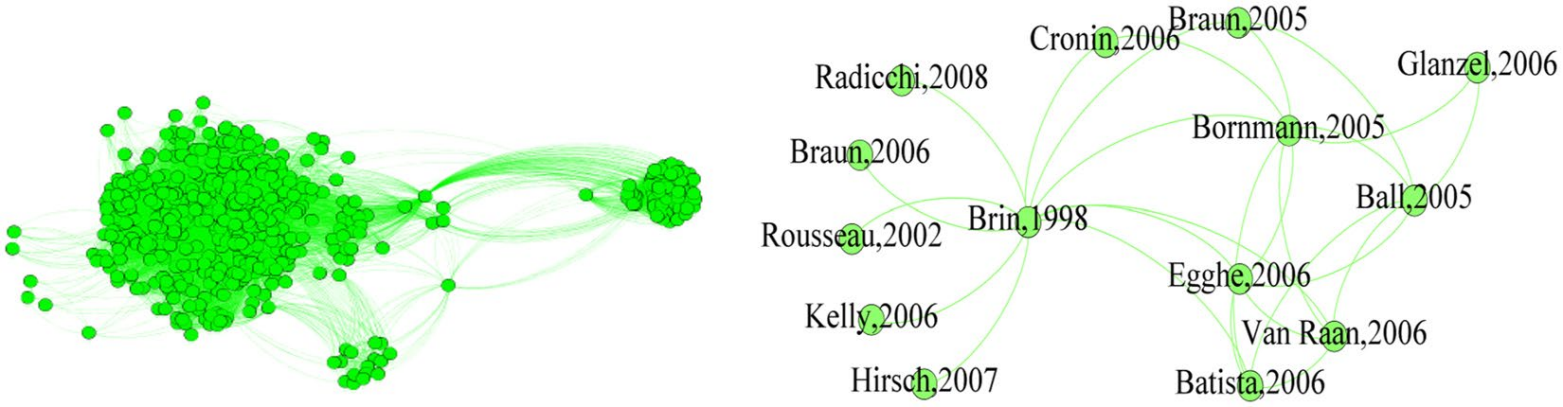
The *h*-backbone of the co-citation network.

Figure 1 shows the procedure for identifying the *h*-backbone in a co-citation network. The left side shows the original network and the right its *h*-backbone.

Figure 1 shows both highly-cited papers, such as Egghe’s paper in 2006 and Ball’s in 2005, and bridge papers that connect related research topics, such as Brin’s article in Computer Networks & ISDN Systems that provides a foundation for many other articles that combine later web search engine design and *h*-index research. Table 2 provides structural information and lists all of the nodes in the *h*-backbone that form the core of the weighted network. The percentages of edges and nodes in the *h*-backbone of the co-citation network vs. the total are 0.08% and 2.47%, respectively.

**Table 2:** All *h*-backbone in the co-citation network.

Author(s)	Title	Journal	Year
Egghe L	Theory and Practice of the g-Index	Scientometrics	2006
Ball P	Index aims for fair ranking of scientists	Nature	2005
Bornmann L, Daniel H D	Does the h-index for ranking of scientists really work?	Scientometrics	2017
Braun T, Glänzel W, Schubert A	A Hirsch-type index for journals	Scientometrics	2006
Batista P D, Campiteli M G, Kinouchi O	Is it possible to compare researchers with different scientific interests?	Scientometrics	2006
Van Raan A F J	Comparison of the Hirsch-index with standard bibliometric indicators and with peer judgment for 147 chemistry research groups	Scientometrics	2006
Braun T, Glänzel W, Schubert A	A Hirsch-type index for journals	Scientometrics	2006
Hirsch J E	Does the h index have predictive power?	Proceedings of the National Academy of Sciences of the United States of America	2007
Kelly C D, Jennions M D	The h index and career assessment by numbers	Trends in Ecology & Evolution	2006
Blaise Cronin, Lokman Meho	Using the h‐index to rank influential information scientistss	Journal of the Association for Information Science and Technology	2006
Glänzel W	On the h-index - A mathematical approach to a new measure of publication activity and citation impact	Scientometrics	2006
Brin S, Page L	The anatomy of a large-scale hypertextual Web search engine ☆	Computer Networks & ISDN Systems	1998
Filippo Radicchi, Santo Fortunato, Claudio Castellano	Universality of Citation Distributions: Toward an Objective Measure of Scientific Impact	Proceedings of the National Academy of Sciences of the United States of America	2008
Rousseau R	Lack of standardisation in informetric research, Comments on “Power laws of research output	Scientometrics	2002

**Figure 2:**
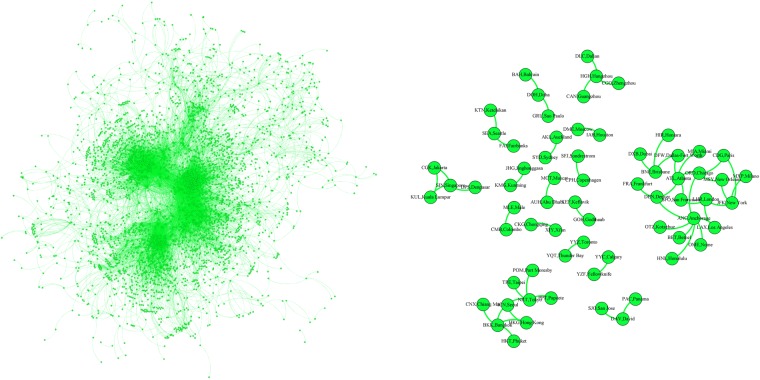
The *h*-backbone of the open flight network.

Figure 2 shows the *h*-backbone of the open flight network. On the left side is the image of the original network and on the right is its *h*-backbone.

In the original open flight network, a node is an airport labeled by its IATA code. To clarify the information, we add the name of the city to the IATA code.

Using the *h*-backbone network we identify the airports that structurally and functionally are most important, e.g., “Chicago-ORD,” which is one of the world’s biggest passenger airports, and “Anchorage-ANC,” which is one of the world’s busiest cargo airports. We evaluate airport performance in terms of passengers, cargo (freight and mail), and aircraft movement. Table 3 supplies examples of important *h*-backbone nodes according to the ACI 2012 World Annual Traffic Report (WATR). The percentages of *h*-backbone edges and nodes in the open flight network vs. the total are 0.30% and 1.96%, respectively.

**Table 3:** Selected representative nodes of the *h*-backbone in the open flight network.

Continent	Airport, City	Typical characteristics
Asia	HKG, Hong Kong	1st in cargo
	ICN, Seoul	5th in cargo
	CGK, Jakarta	9th in passengers
	NRT, Tokyo	10th in cargo
Europe	LHR, London	3rd in passengers
	CDG, Paris	7th in passengers
	FRA, Frankfurt	9th in cargo
North America	ATL, Atlanta	1st in passengers /cargo/movements
	ORD, Chicago	2nd in movements
	LAX, Los Angeles	3rd in movements
	ANC, Anchorage	4th in cargo

Here the airport importance is determined by combining its business in cargo and passengers and its movements. Thus the *h*-backbone quantifies its importance.

**Figure 3:**
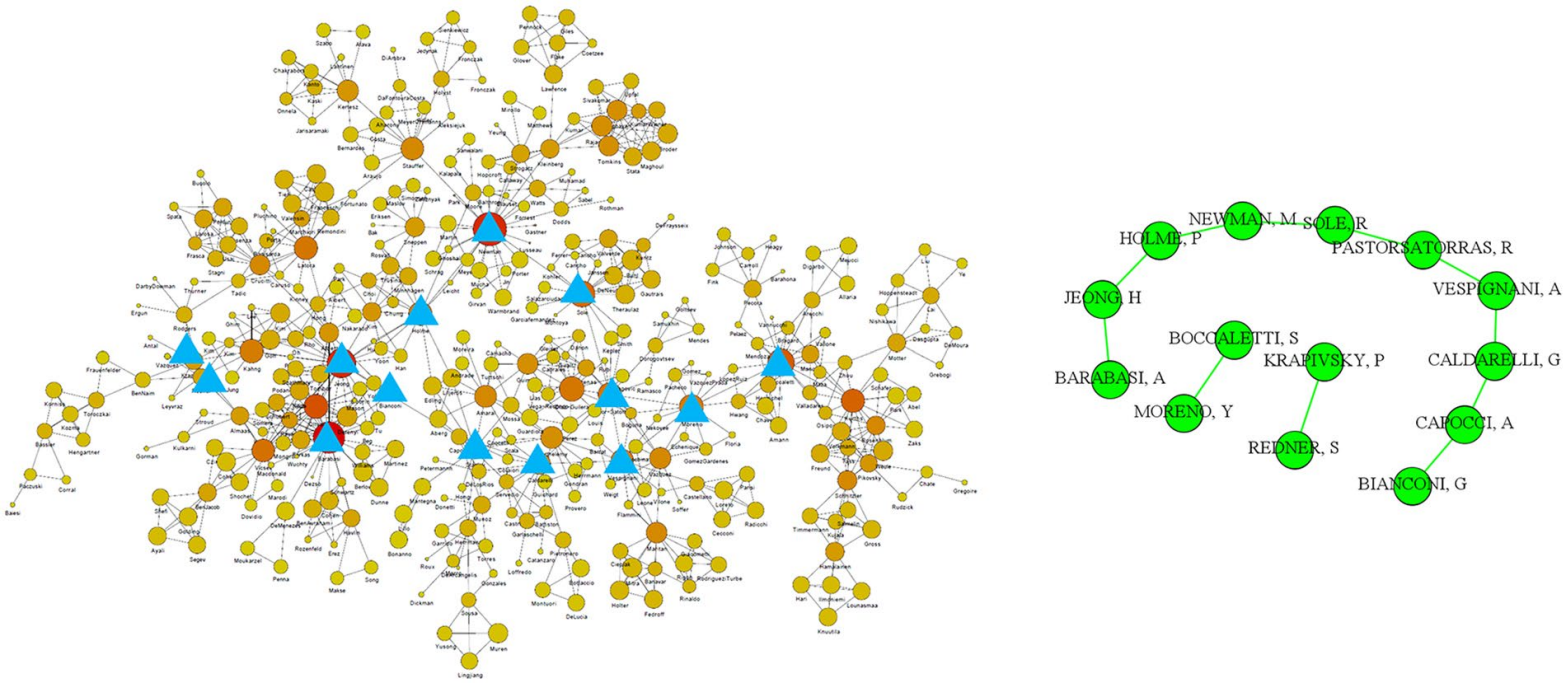
The h-backbone of the coauthorship in network science.

Figure 3 shows the *h*-backbone of coauthorship in network science publishing. On the left side is an image of the original network^24^ in which only the largest component of the resulting network is shown. On the right is the *h*-backbone of the entire network. The blue triangles on the left are the nodes in the *h*-backbone. Note that these *h*-backbone nodes are important in the original network. The percentages of edges and nodes in the *h*-backbone vs. the total are 0.9% and 0.5%, respectively.

These three cases show that we can identify an *h*-backbone in a weighted network, and that with fewer than 1% edges and 3% nodes the *h*-backbone is a core structure in the weighted network. This approach effectively locates and extracts the structurally and functionally important edges with adjacent nodes in weighted networks.

## Discussion

**Figure 4:**
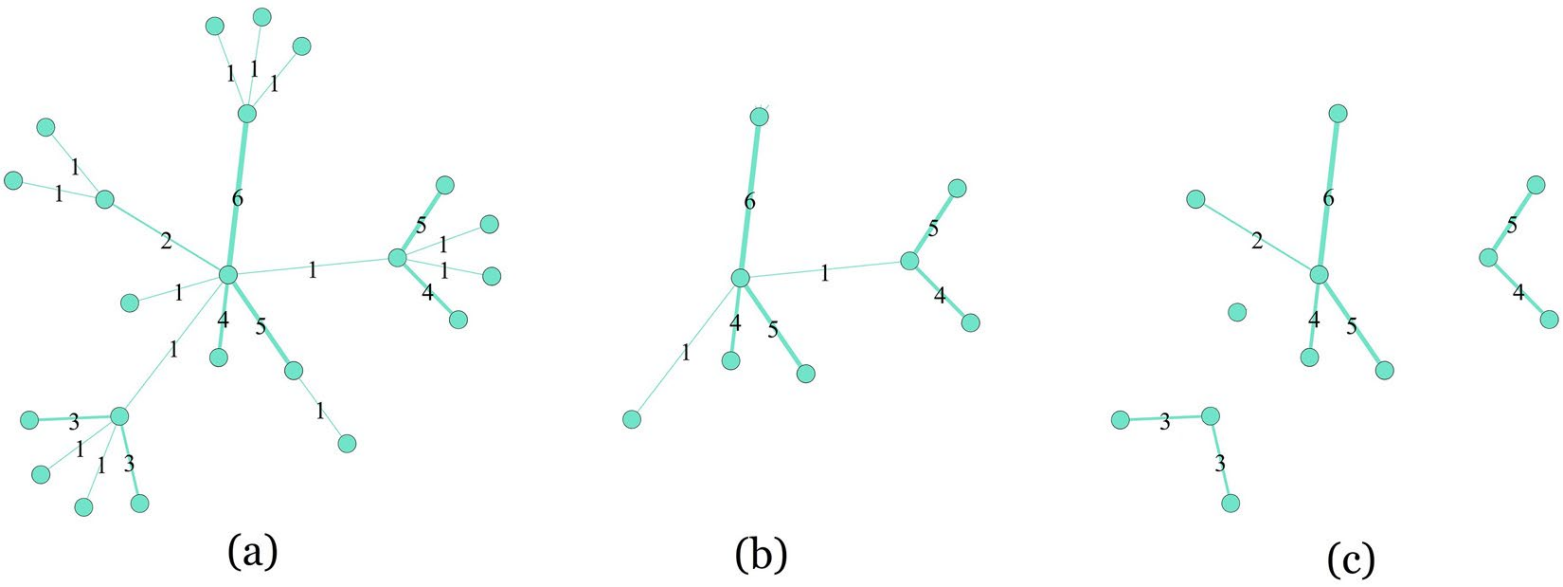
A comparison of the *h*-backbone and the Serrano backbone, (**a**) The original network, where the number of an edge represents its weight (**b**) The *h*-backbone. (**c**) A possible result of the Serrano backbone^10^.

Unlike that found in other backbone approaches^10–12^, the structure of the *h*-backbone is unique in each network. In the Serrano approach, because the adjacent edges in some nodes are assumed to be more significant, they are assigned to the backbone. This “significance” is determined using a “disparity filter” with a variable α that strongly affects how many edges or nodes remain in the backbone. In the *h-*backbone algorithm, the number of edges remaining in the *h*-backbone is determined solely by network characteristics, i.e., edge weight (*h*-strength) and network structure (*h*-bridge). In addition, the *h*-backbone algorithm is highly efficient, and it preserves the small number of edges and nodes that carry important information. In addition, because the *h*-backbone focuses on edges rather than nodes, it retains more structural characteristics. As a result, there are no isolated nodes in the *h*-backbone, and every node is connected to at least one other node. Figure 4 shows a comparative example.

Table 4 shows a computed numerical comparison of the *h*-backbone and the Serrano backbone in three real-world networks.

**Table 4:** Comparative results with overlap ratios of the Serrano backbone and h-backbone.

α	Class	Co-citation network	Open flight network	Coauthorship network
α = 0.01	node	8(100.0%)	39(56.4%)	17(41.2%)
	edge	9(100.0%)	28(39.3%)	10(30.0%)
α = 0.05	node	11(90.9%)	248(20.6%)	67(14.9%)
	edge	22(63.6%)	266(12.4%)	44(6.8%)

In Table 4, the number represents the amount of nodes or edges corresponding to the network. The number in parentheses stands for the percentage of nodes or edges overlapped by the h-backbone, which is the value of the number of nodes or edges both in Serrano backbone and h-backbone divided by the number of nodes or edges in Serrano backbone.

Note that the Serrano backbone requires the artificial parameter α. When this parameter changes, the number of network nodes and edges changes drastically. When α = 0.01, the similarity between the two backbones exceeds 30%, and in one case there is a complete 100% overlap (the co-citation network). When α = 0.05, the similarity is less, in part because the number of edges preserved by the *h*-backbone is smaller than those by the Serrano backbone.

Unlike those in the current literature, the *h*-backbone needs no parameter to adjust the size of the resulting backbone, and thus the *h*-backbone of each network is uniquely determined. Using the *h*-backbone method eliminates artificial interference in the process of backbone extraction.

Both the connected and unconnected *h*-backbones are determined by the original structure of the network. In our examples, the *h*-backbone of the co-citation network is connected and the *h*-backbone of open flight network is unconnected.

In general, if we assume that the *h*-backbone has *m* edges and *n* nodes, with the *h*-bridge and *h*-strength of h_b_ and h_s_ respectively, the number of edges in the *h*-backbone will be fewer than or equal to h_b_ + h_s_, and the number of nodes in the *h*-backbone will be fewer than or equal to 2(h_b_ + h_s_). Because one edge links two nodes, *m* < *n*. Thus

1 $${h}_{b}+{h}_{s}\le m < n\le 2({h}_{b}+{h}_{s}).$$

The structure of *h*-backbones varies from network to network, and because of this complexity we have not attempted to provide a mathematical proof for the *h-*backbone, which limits our efforts, but recent research^28^ has demonstrated the relation between the *h-*index and the coreness. The *h-*backbone combines the structural importance of the *h-*bridge with the functional importance of the *h-*strength, and thus it retains both structural and functional core interactions.

## Conclusion

We have introduced a method of finding the *h*-backbone, which is a core structure in weighted networks. This core network structure of edges and adjacent nodes is important both structurally and functionally, and our method can be used to simplify complex weighted networks. Because the *h*-backbone integrates core edges with adjacent nodes, the important information of the weighted network is retained. Unlike previous backbones, the *h*-backbone is a unique core network structure.

The *h*-backbone methodology can be generalized to other weighted networks. Currently, our case study addresses only undirected weighted information networks, leaving directed weighted and heterogeneous and multilayer weighted networks^29^ for future research. Dynamic issues are also left for future study.

## Method

A network (graph) consists of nodes (vertices) and edges (links)^30,31^. When nodes and edges represent information-related and society-related objects, we designate the two systems information and social networks, respectively.

Theoretically, betweenness centrality is a measure of centrality in a graph based on shortest paths. There are node betweenness and edge betweenness, and we focus on edge betweenness because its centrality quantifies the number of times an edge acts as a bridge in the shortest path between two nodes. Introduced by Linton Freeman^27^, the betweenness centrality of a node is the number of these shortest paths that pass through it. The edge betweenness of an edge can be similarly defined^28^.

In a given network, the edge betweenness of an edge *v* in a network G = (V,E) is defined

2 $$eb(v)=\sum _{s\ne v\ne t}\frac{{\sigma }_{st}(v)}{{\sigma }_{st}},$$

where σ_st_ is the total number of shortest paths from node *s* to node *t* and σ_st_ (*v*) is the number of those paths that pass through edge *v*.

Edge betweenness quantifies the structural importance of a network edge. The edge with a higher edge betweenness often acts as a bridge to transmit information. Note, by definition, in a network of *N* nodes, the maximum edge betweenness of a given edge is *N* × (N-1), i.e., the greater the number of nodes in a network, the larger the edge betweenness of most of the edges. Thus we introduce a new measurement, the *bridge*, which we obtain by dividing the edge betweenness with the number of all nodes N,

3 $$b(v)=\frac{eb(v)}{N}.$$

After we calculate the bridge for all edges, we rank them using an *h*-index approach.

### Definition 1. h-bridge

The h-bridge **(**h_b_**)** of a network is equal to h_b_, if h_b_ is the largest natural number such that there are h_b_ links, each with bridge at least equal to h_b_ in the network.

We also define *h*-strength^20^.

### Definition 2. h-strength

The h-strength **(**h_s_**)** of a network is equal to h_s_, if h_s_ is the largest natural number such that there are h_s_ links, each with strength at least equal to h_s_ in the network.

Because the *h-*bridge quantifies the structurally important edges connecting the network, and the *h*-strength characterizes the core edges of a network in terms of link strengths, we can obtain the core backbone structure by combining them.

### Definition 3. The h-backbone

An h-backbone of a network is a core sub-network consisting of all edges with strengths larger than or equal to the h-bridge or the h-strength in the network, together with their adjacent nodes.

**Figure 5:**
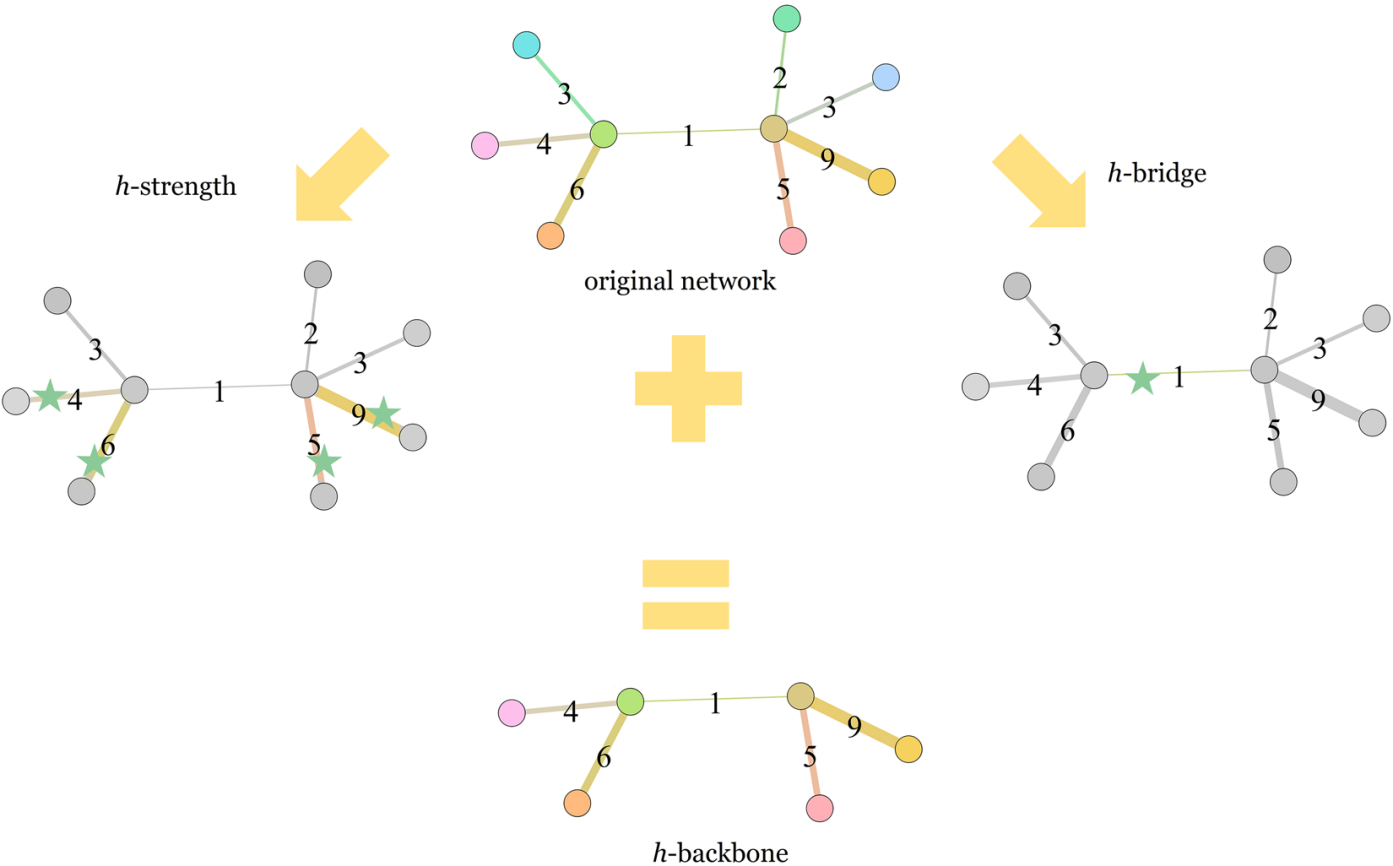
Extracting the *h*-backbone in a weighted network (The pentagram at the edge indicates that this edge is preserved in the step).

In a weighted network the algorithm for extracting the *h*-backbone has three steps (Fig. 5).

Step 1: Find the edges with a bridge higher than or equal to the *h*-bridge;

Step 2: Find the edges with a weight higher than or equal to the *h*-strength;

Step 3: Identify the *h*-backbone by merging the edges of Step 1 and 2 and adding their adjacent nodes.
